# Lateral lumbar interbody fusion after reduction using the percutaneous pedicle screw system in the lateral position for Meyerding grade II spondylolisthesis: a preliminary report of a new lumbar reconstruction strategy

**DOI:** 10.1186/s12891-020-03935-6

**Published:** 2021-01-05

**Authors:** Masanari Takami, Ryo Taiji, Motohiro Okada, Akihito Minamide, Hiroshi Hashizume, Hiroshi Yamada

**Affiliations:** Department of Orthopaedic Surgery, Wakayama Medical University, 811-1 Kimiidera, Wakayama, 641-8510 Japan

**Keywords:** Meyerding grade II spondylolisthesis, Lateral lumbar interbody fusion, Percutaneous pedicle screw, Minimally invasive spine surgery, Lateral position, New surgical technique

## Abstract

**Background:**

Utilization of a cage with a large footprint in lateral lumbar interbody fusion (LLIF) for the treatment of spondylolisthesis leads to a high fusion rate and neurological improvement owing to the indirect decompression effect and excellent alignment correction. However, if an interbody space is too narrow for insertion of an LLIF cage for cases of spondylolisthesis of Meyerding grade II or higher, LLIF cannot be used. Therefore, we developed a novel strategy, LLIF after reduction by the percutaneous pedicle screw (PPS) insertion system in the lateral position (LIFARL), for surgeons to perform accurate and safe LLIF with PPS in patients with such pathology. This study aimed to introduce the new surgical strategy and to present preliminary clinical and radiological results of patients with spondylolisthesis of Meyerding grade II.

**Methods:**

Six consecutive patients (four men and two women; mean age, 72.7 years-old; mean follow-up period, 15.3 months) with L4 spondylolisthesis of Meyerding grade II were included. Regarding the surgical procedure, first, PPSs were inserted into the L4 and L5 vertebrae fluoroscopically, and both rods were placed in the lateral position. The L5 set screws were fixed tightly, and the L4 side of the rod was floated. Second, the L4 vertebra was reduced by fastening the L4 set screws so that they expanded the anteroposterior width of the interbody space. At that time, the L4 set screws were not fully tightened to the rods to prevent the endplate injury. Finally, the LLIF procedure was started. After inserting the cage, a compression force was added to the PPSs, and the L4 set screws were completely fastened.

**Results:**

The mean operative time was 183 min, and the mean blood loss was 90.8 mL. All cages were positioned properly. Visual analog scale score and Oswestry disability index improved postoperatively. Bone union was observed using computed tomography 12 months after surgery.

**Conclusion:**

For cases with difficulty in LLIF cage insertion for Meyerding grade II spondylolisthesis due to the narrow anteroposterior width of interbody space, LIFARL is an option to achieve LLIF combined with posterior PPS accurately and safely.

**Trial registration:**

UMIN-Clinical Trials Registry, UMIN000040268, Registered 29 April 2020, https://upload.umin.ac.jp/cgi-open-bin/ctr/ctr_view.cgi?recptno=R000045938

## Background

**Fig. 1:**
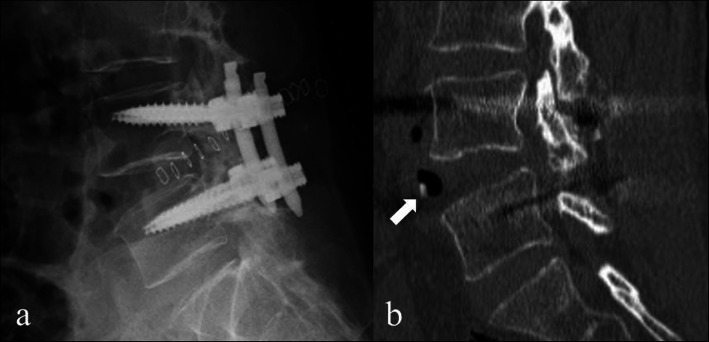
Failure of extreme lateral interbody fusion (XLIF) for a patient with L4 spondylolisthesis. L4–5 fusion surgery, including XLIF, is planned for a 58-year-old woman with L4 spondylolisthesis. However, the anterior longitudinal ligament (ALL) is injured during the XLIF procedure; hence, XLIF is abandoned because it is impossible to fix a cage. Postoperative radiography reveals that the interbody space at the L4/5 level expanded abnormally **a**. After surgery, a bony endplate fracture (white arrow) associated with the ALL tear is detected on computed tomography **b**

Spondylolisthesis (SL) is a common disease, and surgical treatment is sometimes selected when patients complain of leg or back pain. Posterior lumbar interbody fusion (PLIF) and transforaminal lumbar interbody fusion are conventional surgical methods for treating SL [1]. Lateral lumbar interbody fusion (LLIF) has been developed recently [2]. Subsequently, the use of minimally invasive spinal surgery involving LLIF combined with posterior percutaneous pedicle screw (PPS) fixation [3] for degenerative lumbar disease was reported. Utilization of a cage with a large footprint in LLIF leads to a high fusion rate [4–6] and neurological improvement because of the indirect decompression effect and excellent alignment correction [2, 7]. This surgical strategy has been adopted in patients with SL, and good clinical and radiological results have been demonstrated [8–17]. However, if patients, especially Asians with small physiques, have grade II or higher SL according to the Meyerding classification [18], there is a disadvantage in that it is difficult to insert the large cage into a narrow interbody space (Fig. 1a, b). Therefore, we developed a novel surgical strategy of lateral lumbar interbody fusion after reduction by the posterior percutaneous pedicle screw system in the lateral position (LIFARL) for such patients. To the best of our knowledge, this is the first description of such a strategy. The current study aimed to introduce our novel surgical strategy and present preliminary clinical and radiological results.

## Methods

### Study design and patients

The study design was approved by the institutional review board of our university before the initiation of the study. The patients or their family members were informed that the patient data would be submitted for publication, and their written consent was obtained.

We performed extreme lateral interbody fusion (XLIF®, NuVasive, San Diego, CA, USA) combined with PPS (Reline MAS system®, NuVasive) in the lateral position for 15 patients with lumbar diseases since June 2018 and developed our novel strategy and have been using it since September 2018. Six consecutive patients (four men and two women) who underwent fusion surgery by this method in our institute for Meyerding grade II SL with leg or low back pain were included in this study.

### Surgical procedure

**Fig. 2:**
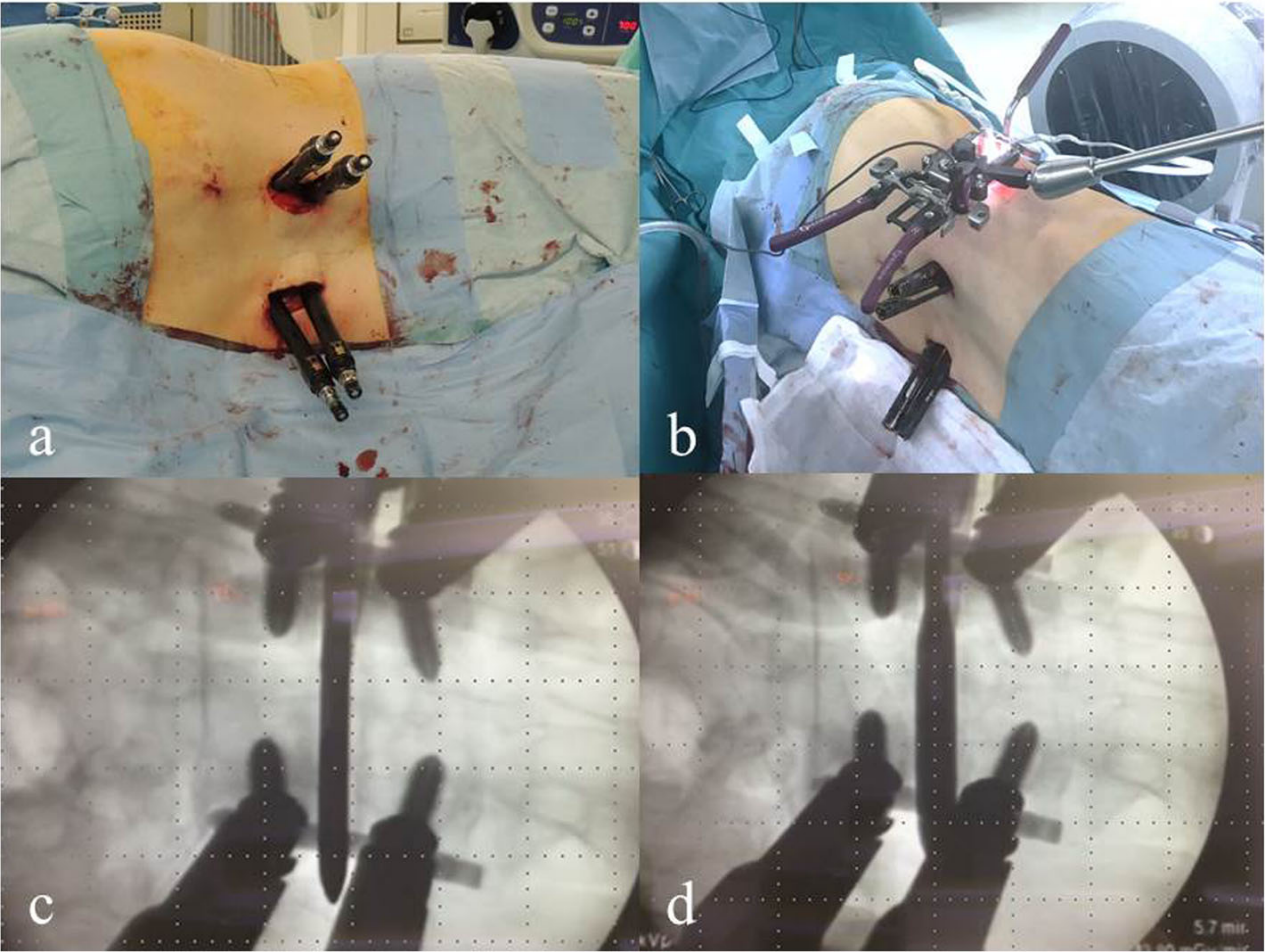
Intraoperative image in the lateral position and fluoroscopic imaging. Surgery is started by inserting the percutaneous pedicle screws with the patient in the lateral position **a**. Extreme lateral interbody fusion is performed after reduction by using the percutaneous pedicle screws system **b**. During extreme lateral interbody fusion, fluoroscopy is used to insert the XLIF Paddle Sizer® **c**, and when the Paddle Sizer is rotated, there is some movement between the L4 and L5 vertebrae **d**. This helps to prevent endplate injury during cage insertion

The following new surgical strategy was described as a case of L4–5 fusion surgery for SL of the L4 vertebra. After placing the patient in the lateral position, XLIF and posterior PPS were used simultaneously. First, PPSs were inserted into the L4 and L5 vertebrae fluoroscopically (Fig. 2a), and rods on both sides of appropriate lengths were placed. The final fixation of the L5 set screws on both sides was done, and the L4 side of the rod was floated. Next, the L4 vertebra was reduced to some extent by fastening the L4 set screws on both sides through the extender such that they expanded to the anteroposterior width of the interbody space. Using this operation, Meyerding grade changed from II to I, and the cage could be safely inserted. At this time, the L4 set screws were not fully tightened to the rods (i.e., movement between the L4 PPSs and rods remained). The anteroposterior space in which the XLIF cage would be inserted was expanded by this reduction. Finally, XLIF was started, and disc curettage was conducted after the retractor was placed in the lateral position (Fig. 2b). At this time, motion between the L4 and L5 vertebrae was confirmed under fluoroscopy (Fig. 2c, d), which helped to prevent endplate injury upon insertion of the XLIF cage. After reducing the L4 vertebra again by tightening the L4 set screws if necessary, the XLIF cage was inserted. Reduction of the L4 vertebra was never performed after cage insertion to prevent endplate injury. After the bending of the surgical table was restored, a compression force was added to the PPSs, and both L4 set screws were completely fastened to finish the surgical procedures.

### Evaluation of clinical variables

The operative time and estimated blood loss were evaluated. Complications and radiological findings were evaluated. Measurements of slippage, treated intervertebral heights, evaluation of endplate injury during surgery, malposition of the pedicle screws and screw loosening, inserted cage position in the interbody space, bone union based on the Proietti classification [4], and cage subsidence (over 2 mm) were included in the radiological investigations. Regarding radiological evaluations, bone union was assessed using computed tomography (CT) 12 months after surgery, and the other factors were evaluated by X-rays at the final observation. Regarding clinical results, the Oswestry disability index (ODI) and low back and leg pain based on visual analog scale (VAS) score were evaluated at the final observation.

## Results

### Clinical and radiological outcomes

Mean age was 72.7 years (range, 52–83 years), and the mean follow-up period was 15.3 months (Table 1).

**Table 1:** Clinical characteristics of patients

Case	Level of Spodylolisthesis	Meyerding classification	Fusion level	Follow-up period (mos.)
1	L4	Grade II	L4–5	19
2	L4	Grade II	L4–5	19
3	L4	Grade II	L3–5	18
4	L4	Grade II	L4–5	14
5	L4	Grade II	L4–5	12
6	L4	Grade II	L3–5	10

**Fig. 3:**
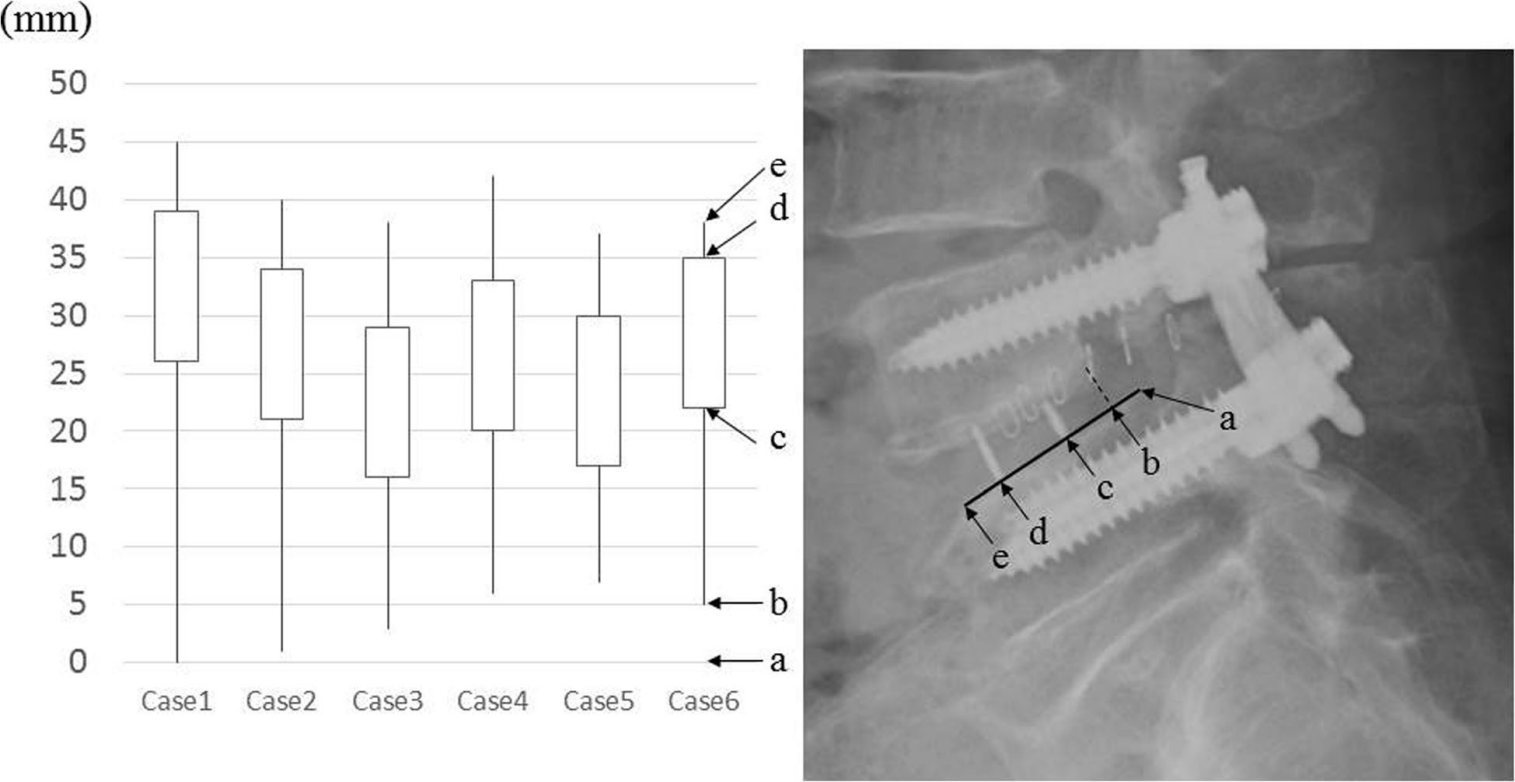
Evaluation of accuracy of inserted cage in extreme lateral interbody fusion. “**a**” is a point of the posterior edge of the L5 vertebra on the upper bony endplate (solid black line) in the postoperative lateral image. “**b**” is an intersection point with a perpendicular line (dashed line) from the posterior edge of the L4 vertebra to the black line. “**c**” is the point of the posterior marker of the cage, and “**d**” is the same point but anteriorly. “e” is the point of the anterior edge of the upper bony endplate of the L5 vertebra. The figure on the right shows the position of the inserted cage. Because the markers are placed 3 mm from the end of the cage, all cages are appropriately inserted into an interbody space

The following data are presented as mean ± standard deviation. The mean operative time was 183.0 ± 40.3 min, and the mean estimated blood loss was 90.8 ± 68.2 mL (Table 2). No complications occurred except in one patient who developed temporary quadriceps weakness for 2 months. Regarding the radiological evaluations, the average slippage amount improved from 12.7 ± 0.8 mm preoperatively to 3.7 ± 2.8 mm at the final observation. The percentage of slippage improved from 33.2 ± 2.4% preoperatively to 9.5 ± 7.3% at the final observation. The height between the vertebrae improved from 3.3 ± 2.7 mm preoperatively to 8.7 ± 0.5 mm at the final observation. There were no cases of endplate injury during surgery, malposition of the pedicle screws, or screw loosening. All cages were inserted properly (Fig. 3). Regarding bone union, type IV (bone bridges inside one of the two internal spaces of the cage and on one side outside of the cage), and type V (bone bridges inside one of the two internal spaces of the cage and on both sides outside of cage) according to the Proietti classification was found in three and three cases, respectively. No cage subsidence was found. The mean ODI improved from 44.2 ± 4.8 preoperatively to 23.7 ± 15.3 postoperatively. The mean VAS score for low back pain improved from 67.5 ± 7.7 mm preoperatively to 21.8 ± 17.8 mm postoperatively, and the mean VAS score for leg pain improved from 65.8 ± 18.3 mm preoperatively to 19.8 ± 17.8 mm postoperatively. In case 2, pre-ODI was nearly equal to post-ODI because it was evaluated immediately before total knee arthroplasty due to his right knee osteoarthritis. However, his low back and leg pain, as indicated by VAS, have improved. No patient required additional decompression surgery postoperatively.

**Table 2:** Clinical and radiological results. Regarding clinical results, measurements of vertebral slippage and disc heights using lateral lumbar radiography before and after surgical correction, and low back and leg pain using VAS score and ODI before surgical treatment and at the latest clinical follow-up were performed. Bone union pattern by Proietti classification was evaluated 12 months after surgery, and cage subsidence was examined at the final observation

Case	Op. time (min.)	EBL (g)	APW (mm)	Slippage (mm) (%)		DH (mm)		Low back pain in VAS		Leg pain in VAS		ODI		Bone fusion by Proietti classification	Cage subsidence
				Pre-op	Post-op	Pre-op	Post op	Pre-op	Post-op	Pre-op	Post-op	Pre-op	Post-op		
1	203	130	25	12 (31.6%)	0 (0.0%)	1	8	70	22	50	23	46	26	IV	No
2	202	205	24	12 (30.0%)	1 (2.5%)	6	9	71	18	100	15	47	51*	IV	No
3	163	30	21	13 (34.2%)	3 (7.9%)	4	9	73	10	72	9	48	10	IV	No
4	162	30	20	13 (34.2%)	6 (14.3%)	1	9	73	10	60	20	48	9	V	No
5	127	55	17	12 (32.4%)	7 (18.9%)	7	8	53	57	55	52	38	26	V	No
6	241	95	21	14 (36.8%)	5 (13.1%)	1	8	65	14	58	0	38	20	V†	No

*EBS* Estimated blood loss, *APW* Anteroposterior width of interbody space at spondylolisthesis preoperatively, *DH* Disc height, *ODI* Oswestry disability index, *This item was evaluated immediately before total knee arthroplasty due to his right knee osteoarthritis, ^†^This evaluation was performed 10 months after surgery

### Representative case

**Fig. 4:**
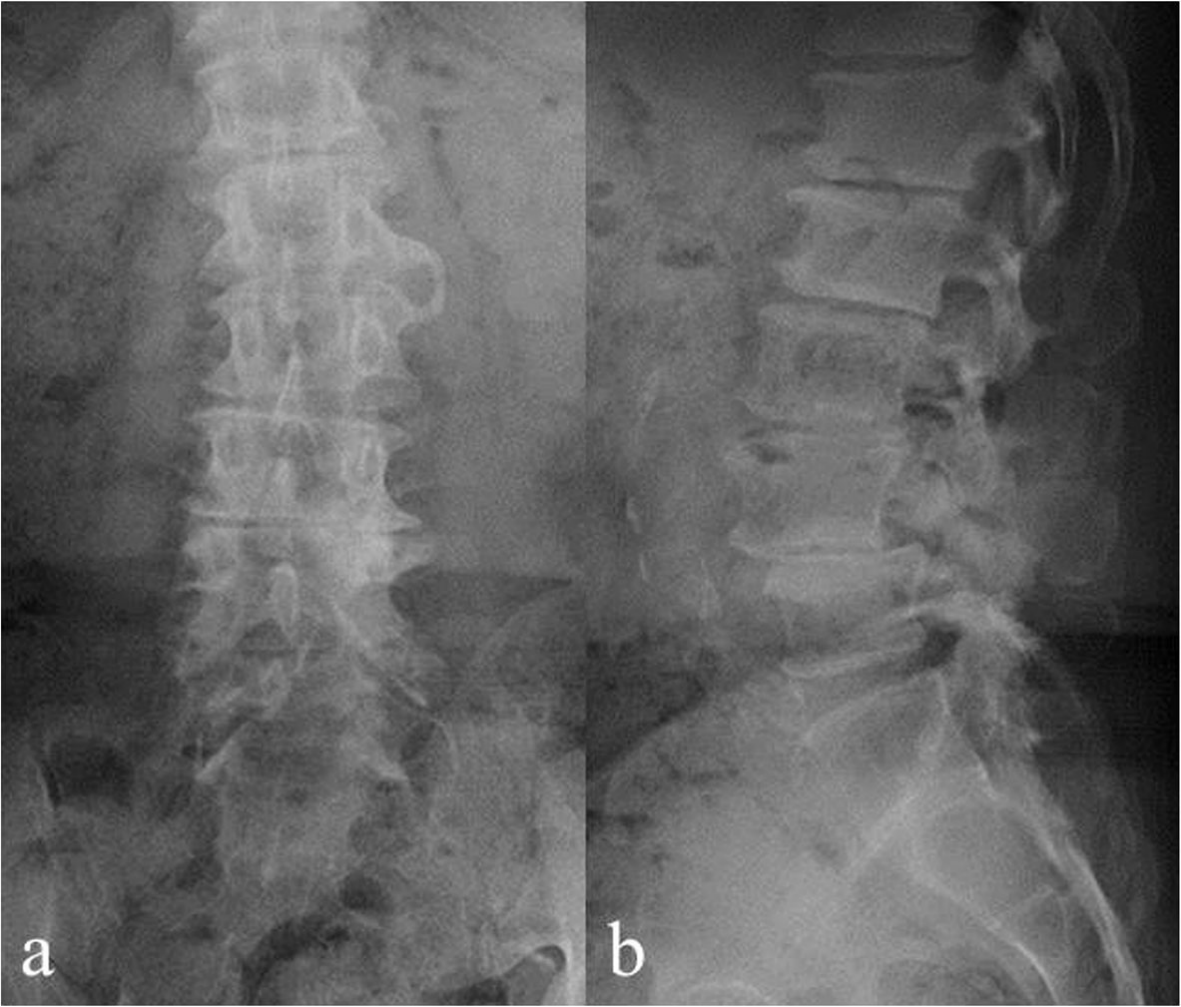
Preoperative radiographic imaging. Preoperative radiographic coronal image **a** and radiographic lateral image **b** showing Meyerding grade II spondylolisthesis

**Fig. 5:**
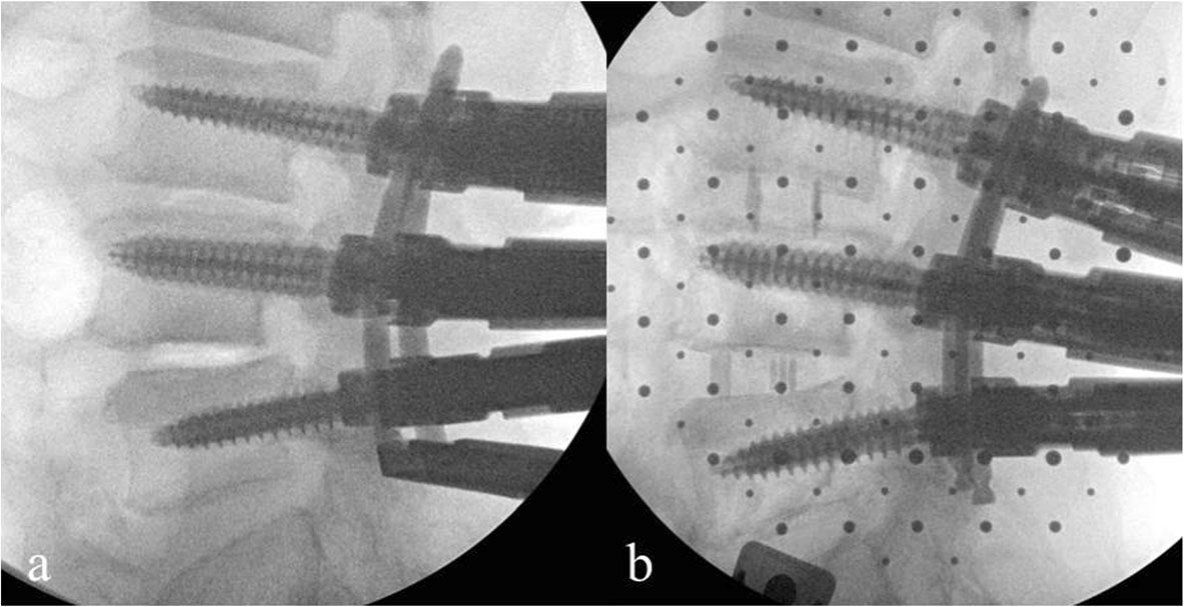
Intraoperative fluoroscopic imaging during slipped vertebra reduction prior to extreme lateral interbody fusion. Fluoroscopic lateral image after reduction is performed by pulling the rods backward, and the extender shows an increase of anteroposterior interbody space at the L4/5 level, which means that it was easy to insert the lumbar interbody fusion cage safely and accurately **a**. Fluoroscopic lateral image after extreme lateral interbody fusion showing more reduction of the L4 vertebra **b**

**Fig. 6:**
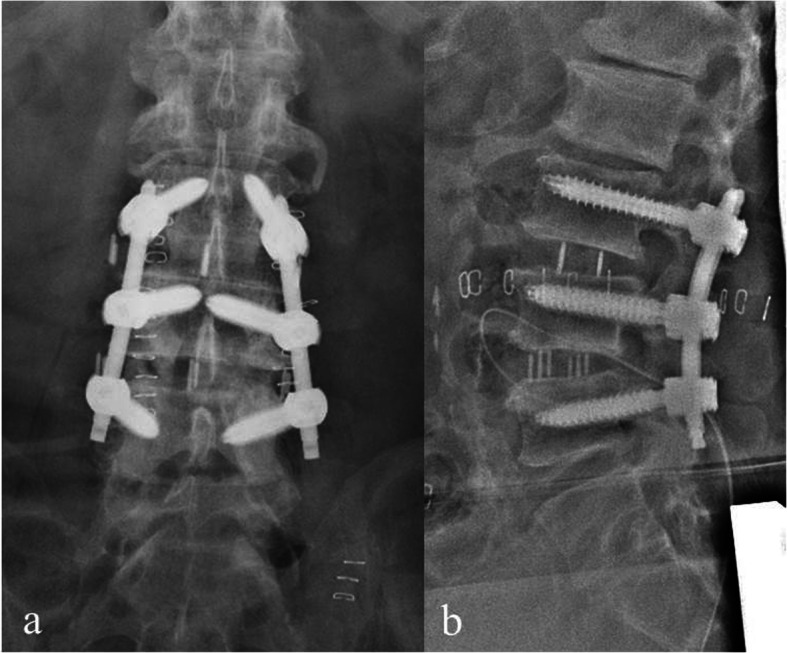
Postoperative radiographic imaging. Postoperative radiographic images **a**, **b** showing good alignment

A 64-year-old man was diagnosed with degenerative SL of the L4 vertebra with instability and simple lumbar spinal stenosis at the L3/4 levels (Fig. 4a, b). Spinal fusion surgery at the L3–5 levels was performed (Fig. 5 & 6a, b, a, b). The operative time was 163 min, and blood loss was 30 mL. At 18 months postoperatively, the patient’s lumbar and leg pains disappeared. Postoperative magnetic resonance imaging revealed that the spinal canal had expanded enough by indirect decompression. Type IV bone union according to the Proietti classification was observed using CT at 12 months postoperatively. No implant-related complications occurred at the final observation.

## Discussion

This is the first description of the novel strategy to perform XLIF combined with posterior PPS for patients with Meyerding grade II SL. Conventional XLIF combined with PPS procedure for such patients is contraindicated because of the narrow anteroposterior width of the interbody space. Therefore, we developed the current surgical strategy of the reduction by the posterior PPS system in the lateral position prior to LLIF procedure.

Recently, it was reported that LLIF combined with posterior PPS for SL was associated with good clinical and radiological outcomes [8–17]. This combined procedure showed lower blood loss and muscle damage than other types of lumbar interbody fusion [9, 13, 16, 17]. Additionally, it has been indicated that this combined procedure is associated with a higher correction rate and better mechanical stability [12], higher bone union rate [8, 19], and lower complication rate [8, 11, 19] than existing lumbar interbody fusion because a cage with a larger footprint is used. Good correction rates and clinical results were reported for isthmic SL by using the same procedure [14]. Therefore, this strategy may not only be a minimally invasive method but also an effective method of lumbar reconstruction surgery for SL. Moreover, another advantage was reported that performing LLIF with PPS with patients in the single lateral decubitus position saved time and money and improved operative efficacy [20, 21].

The main concern for surgeons was the occurrence of incidental anterior longitudinal ligament or neurological injury from inserting the cage into a narrow interbody space in patients with severe SL. The minimum anteroposterior width of the currently available cage for LLIF is 18 mm in Japan. Therefore, if the width was less than about 20 mm, the surgeon cannot insert the cage using the conventional surgical procedure of LLIF theoretically. In some cases, the position may be slightly reduced. Nevertheless, it is considered dangerous to place the LIF cage first before reduction with PPS in patients with Meyerding grade II spondylolisthesis because the posterior nerves are very close to the retractor, and the appropriate range of cage placement is extremely narrow in the actual surgical field even if a slight reduction is obtained in the lateral position (particularly at L4/5 level). The surgeon must avoid both nerve injury and anterior longitudinal ligament injury, and hence, naturally, the surgeon prefers to have some extra space for safe and exact cage placement. We designed our novel surgical strategy to overcome this challenge.

The main limitation of this study was the small sample size and did not include a control group for statistical analysis of effectiveness of our method. The present six cases accounted for over 50% of all the cases who underwent XLIF combined PPS in the lateral position in our institution from 2018. However, in general, the rate of Meyerding grade II or higher is not very high in the SL. Second, the follow-up period may be short to assess the efficacy of this surgical strategy. Strong reduction for severe SL may lead to instability between the vertebrae. Fogel et al. [22] reported that a biomechanical test showed variability in construct stability in the sagittal plane of LLIF combined with PPS. LIFARL aims to achieve safe insertion of the LLIF cage with some reduction, and it is not intended for complete reduction. No cases of pseudarthrosis and implant-related complications occurred, however, it will be necessary to confirm the efficacy of this strategy in a larger number of patients with appropriate follow-up period in the future.

## Conclusion

Conventional XLIF combined with PPS procedure for patients with SL of Meyerding grade II is contraindicated because of the narrow anteroposterior width of the interbody space. However, by using the LIFARL strategy, surgeons have an option to perform LLIF combined with posterior PPS accurately and safely. For patients with such pathology, the minimally invasive LIFARL strategy provides excellent clinical outcomes.

## Data Availability

All data generated or analyzed during this study are included in this published article.

## Abbreviations

LLIF: Lateral lumbar interbody fusion
XLIF: extreme lateral interbody fusion
PPS: Percutaneous pedicle screw
LIFARL: Lateral lumbar interbody fusion after reduction by the posterior percutaneous pedicle screw system in the lateral position
SL: Spondylolisthesis
PLIF: Posterior lumbar interbody fusion
CT: computed tomography
ODI: Oswestry disability index
VAS: Visual analog scale
